# Model-based hierarchical reinforcement learning and human action control

**DOI:** 10.1098/rstb.2013.0480

**Published:** 2014-11-05

**Authors:** Matthew Botvinick, Ari Weinstein

**Affiliations:** Princeton Neuroscience Institute and Department of Psychology, Princeton University, Princeton, NJ 08540, USA

**Keywords:** reinforcement learning, goal-directed behaviour, hierarchy

## Abstract

Recent work has reawakened interest in goal-directed or ‘model-based’ choice, where decisions are based on prospective evaluation of potential action outcomes. Concurrently, there has been growing attention to the role of hierarchy in decision-making and action control. We focus here on the intersection between these two areas of interest, considering the topic of *hierarchical model-based control.* To characterize this form of action control, we draw on the computational framework of hierarchical reinforcement learning, using this to interpret recent empirical findings. The resulting picture reveals how hierarchical model-based mechanisms might play a special and pivotal role in human decision-making, dramatically extending the scope and complexity of human behaviour.

## 1. Introduction

Reinforcement learning (RL) theory has, over recent years, exerted a seismic influence on cognitive science and neuroscience. The inception of this effect was, of course, the discovery that the dynamics of dopamine release, as well as certain dopamine-dependent forms of learning, could be neatly modelled in terms of temporal-difference algorithms for RL (see [1]). As the emergence of this fundamental insight, the role of RL in cognitive science has expanded rapidly. Whereas it was initially treated as a repository of specific computational techniques, RL has come increasingly to provide a general framework for thinking about motivated behaviour and learning in humans and other animals [2].

As its role has broadened in this way, research has gradually moved beyond a monolithic view of RL problems and methods, attending increasingly to some fundamental internal distinctions. An important example is the contrast between model-free and model-based RL [3,4]. In computational terms, *model-free* RL assumes that learning occurs without access to any internal representation of the causal structure of the environment. Rather than building such an internal model, the agent instead simply stores estimates for the expected values of the actions available in each state or context, shaped by a history of direct interaction with the environment. In *model-based* RL, in contrast, the agent does possess an internal model, one that both predicts action outcomes and estimates the immediate reward associated with specific situations. Decisions are made not on the basis of stored action values, but instead through *planning*: the prospective use of the internal model to simulate and compare candidate lines of behaviour.^1^

In the earliest scientific work to leverage concepts from RL, the focus was almost exclusively on the model-free case, to which dopamine function initially seemed most intimately connected. However, attention has subsequently expanded to consider the potential relevance of model-based RL [4,5]. Over the past few years, a series of studies investigating possible roles for model-based RL in human and animal decision-making has renewed interest in the time-honoured problem of planning [6,7] (see [8]).

In this article, we consider the relationship between model-based RL and another form of RL that has also recently become a topic of discussion in cognitive science and neuroscience, namely *hierarchical reinforcement learning* (HRL) [9–11]. The basic idea in HRL is to augment the set of actions available to the agent to include a set of temporally extended multi-action subroutines or skills. Thus, where a non-hierarchical (‘flat’) RL agent might select among simple actions like ‘press the *a* key’ and ‘click the mouse button’, an HRL agent in the same situation might also be in a position to elect to ‘log in to email account’, a skill that would in turn comprise a coordinated sequence of low-level actions.^2^ Introducing such extended, ‘temporally abstract’ actions into RL can have dramatic computational benefits, allowing RL agents to conquer problems too large to solve through flat RL. In recent work, we and others have proposed that the human brain might leverage HRL-like representations and procedures in order to deal tractably with complex real-world decision problems [10,14,15]. Initial research, some of which we shall summarize below, provides significant encouragement for this hypothesis.

Importantly, the computational literature describes both model-free and model-based varieties of HRL. However, as with RL at large, the initial applications of HRL within psychology and neuroscience have focused almost entirely on the model-free case. Our purpose in this article is to argue for the potential relevance of model-based HRL (MB-HRL) to understanding human action. Indeed, we suspect that MB-HRL may hold an indispensable key to understanding the remarkable scope, efficacy and flexibility of human behaviour. As we shall detail, the combination of temporal abstraction with prospective, model-based planning can yield dramatic, synergistic pay-offs, markedly extending a decision-maker's cognitive reach. MB-HRL provides a well-developed computational framework for understanding this synergy, and thus offers a powerful set of conceptual tools for investigating human action.

In what follows, we begin by offering a more concrete introduction to both model-free and MB-HRL, along with a consideration of the special computational advantages of MB-HRL. We then consider empirical evidence suggesting the potential relevance of MB-HRL to human planning, pointing out some key questions for further research.

## 2. Model-based versus model-free hierarchical reinforcement learning

As summarized above, the distinction between model-free and model-based RL lies fundamentally in what information the agent stores in memory. In model-free RL, the agent stores a representation of the *value* associated with individual actions: an estimate of the cumulative reward the agent expects to accrue over the course of future behaviour, when beginning in a particular situation with a particular action. Updates to these values—known collectively as the *value function*—are driven by *reward prediction error* (RPE) signals, generated based on action outcomes experienced during direct interaction with the environment.

By contrast, model-based RL, at least as it has been considered in recent cognitive science research, does not depend on a stored value function. Instead, the agent maintains a two-part internal model. The first part of this dyad, the *transition model*, represents the causal structure of the behavioural environment, supporting predictions concerning (potentially probabilistic) action outcomes. The second part, the *reward model*, represents an estimate of the immediate reward (possibly negative) associated with individual situations or actions. Armed with these two knowledge structures, the decision-maker is in a position to simulate potential courses of action, attach values to their associated outcomes, and thereby choose adaptively among them, that is, to plan.

The model-free/model-based distinction extends directly to the case of HRL. As we have noted, HRL expands the set of actions available to the RL agent to include a set of temporally extended subtasks or subroutines. In the implementation of HRL that we will take as our focus—the *options framework*^3^ introduced by Sutton *et al*. [16]—these temporally abstract actions are referred to as options. Options can be selected for execution, just like low-level (‘primitive’) actions. Once this happens, behaviour is guided by an option-specific *policy*, which dictates the action to be selected in each possible situation or state. Each option is additionally associated with an *initiation set*, defining the situations in which the option can be selected or launched; a *termination function*, which dictates when execution of the option ends; and an option-specific reward function, a *pseudo-reward function*, which attaches a special form of reward to specific outcomes, effectively defining the goals being pursued during execution of the option.

In *model-free HRL* (MF-HRL), just as in model-free ‘flat’ RL, the agent maintains a value function, estimating the long-term cumulative reward associated with specific actions, and updates this based on RPEs. However, the set of actions addressed by this value function now includes not only primitive actions, but options as well. Furthermore, the RPE-based learning mechanism operates not only to shape the agent's overall value function, but also to shape option-specific policies.^4^ Model-free RL thus operates at each level of the agent's action hierarchy.

**Figure 1.**
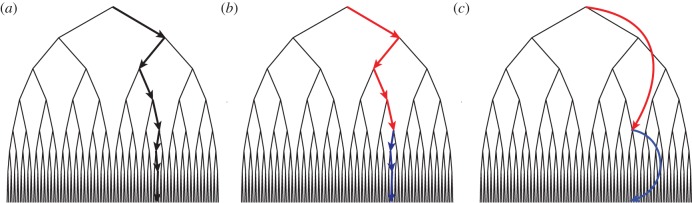
An illustration of how options can facilitate planning. (*a*) A search tree with arrows indicating the pathway to a goal state. A specific sequence of seven independently selected actions is required to reach the goal. (*b*) The same tree and trajectory, the colours indicating that the first four and the last three actions have been aggregated into options. Here, the goal state is reached after only two independent choices (selection of the options). (*c*) Illustration of planning using option models, which allow the ultimate consequences of an option to be forecast without requiring consideration of the lower level steps that would be involved in executing the option. Planning with options effectively reduces the number of decisions needed to reach any depth in the tree. (Adapted from [10].)

In MB-HRL, the agent once again carries a transition model supporting the prediction of action outcomes, as well as a reward model attaching a reward magnitude to each such outcome. As in the flat case, this two-part internal model allows the agent to ‘imagine’ and compare different courses of action, using its internal model in place of the external environment. The difference from flat RL is that, as part of this internal simulation, the HRL agent can select not only primitive-level actions, but also the temporally extended behaviours specified by options (figure 1).

As we shall see, the ability to plan with options can have important repercussions, even if the agent's transition model is flat, allowing outcome predictions only for primitive actions. However, in MB-HRL, there is also the possibility for something more: the agent's internal model can itself be temporally abstract. That is, the agent's transition model can incorporate predictions addressing not only the outcomes of primitive actions, but also the ultimate outcomes brought on by executing options. When enabled to make such predictions, the agent is relieved of the need to simulate each low-level step in the behaviours it is considering. Instead, it can leap directly to the predicted outcomes of entire subtask sequences (see figure 1*c*). We shall refer to this leaping form of MB-HRL as *saltatory MB-HRL*, distinguishing it from non-saltatory MB-HRL, where the transition model is flat, predicting outcomes only for primitive actions.

In order to enable saltatory MB-HRL, the options framework introduces a critical new element, referred to as an *option model* (see [16–18])*.* This knowledge structure bears three key pieces of information about a specific option: how the option will end; how long the option will take to execute and how much reward is likely to be accrued during execution of the option. More formally, each option model specifies, for a particular option, (i) a joint probability distribution over option duration and termination state and (ii) the average cumulative temporally discounted reward expected during execution of the option. For example, an option model for an option *buy-movie-tickets-online* might specify a 95% chance of success in obtaining tickets, a 3% chance of failure due to a sell-out, and a 2% chance of failure due to the website hanging. Along with these outcome predictions, the option model would also specify how long it would typically take to arrive at each outcome, as well as how pleasant the whole online procedure typically is to conduct.

## 3. Model-based hierarchical reinforcement learning: computational pay-offs

**Figure 2.**
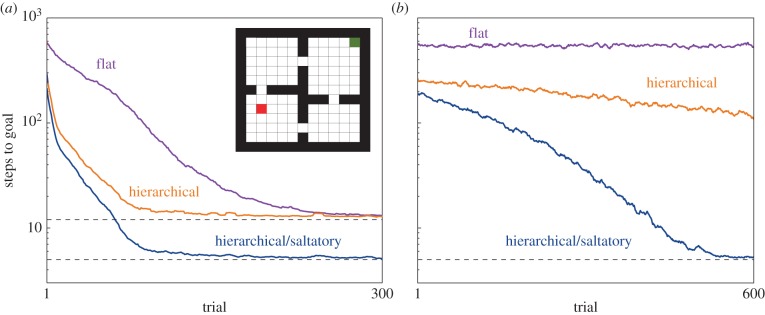
Inset: the rooms task introduced by Sutton *et al*. [16]. The start location is marked in green, the goal location in red. (*a*) Planning performance of three model-based RL agents on the rooms task. Each trial began with the agent at the start location and ended when the agent's planning trajectory reached the goal location. The *y*-axis indicates the number of actions simulated by the agent before the planning trajectory reached the goal. *Flat*: a model-based RL agent using only primitive actions. *Hierarchical*: a MB-HRL agent capable of making predictions only about the outcomes of primitive actions. As noted in the main text, the agent carries a set of options for navigating to the doorways in the rooms domain. *Hierarchical/saltatory*: a MB-HRL agent furnished with option models. The upper dashed line marks the minimum number of primitive actions required to reach the goal; the lower dashed line marks the minimum number of actions using option models. (*b*) Planning performance of the same three agents when labouring under memory limitations. On each step of planning, the agent had a 0.1 probability of ‘losing its place’, triggering an immediate termination of the trial. To facilitate comparison with the data in (*a*), the step-counts shown indicate the number of steps each agent would have taken, had it been allowed to continue to the goal on the relevant trial (see appendix A). All data-series are based on means across 100 simulation runs, smoothed using a 10-step moving average.

The ability to plan hierarchically can have a dramatic impact on planning performance [16,17,19]. To illustrate this, we turn to an example problem that has been frequently employed in the HRL literature. Figure 2 shows the layout of the ‘rooms’ domain [16]. Here, an agent moves among the discrete locations indicated by the grid in the figure, starting from the location marked in green. The objective is to arrive at the goal location (red), where a reward can be collected. Each step carries a small cost, so the best plan is one that follows a shortest path from start to goal.

Figure 2*a* shows the time-course of planning for three different agents. Following a standard approach, planning is modelled here as involving a series of internal simulations, each sampling a trajectory through the problem space. The agent's internal model is used both to project the outcomes of successive actions and to improve the agent's plan based on those projections (see [8] for a general discussion of sample-based planning, and the appendix A of this paper for our specific implementation). The *x*-axis in the figure indexes these internal simulation trials, each of which begins at the start location and continues until the goal is reached. The *y*-axis indicates the number of actions the agent required, on each trial, to reach the goal (mean over 100 replications of the entire planning process). The downward slope of each data-series indicates the speed with which the relevant agent is able to converge on the optimal policy.

The violet data-series in figure 2*a* shows the time-course of planning for a flat RL agent. The orange data-series shows the time-course for a MB-HRL agent. This latter agent carries precisely the same model as the flat agent but is also provided with a set of options, each specifying a policy for reaching one of the doorways in the rooms domain. As the figure makes clear, the ability to ‘try out’ these subgoal-oriented options, alongside primitive one-step actions, yields a dramatic reduction in planning time.

This effect stems from the fact that options *structure* the agent's search among candidate behaviours, guiding that search into channels that fit well with the organization of the problem domain (see [20]). This pay-off is precisely analogous to the one gained by adding temporal abstraction to model-free RL [10]. The only difference is that in MF-HRL the agent learns a policy through direct interaction with the environment, whereas in MB-HRL, the search is conducted using an internal model of the environment.

Planning by way of an internal model has the benefit that it can relieve the agent from having to ‘make mistakes’ in the real world, allowing it to work out a good plan before having to interact directly with the (potentially dangerous) environment. Furthermore, as has been extensively discussed in the recent cognitive science literature, use of an internal model can also allow behaviour to adapt more flexibly to sudden changes in environmental contingencies [3]. These are advantages of model-based RL in general, rather than of MB-HRL *per se*. However, access to an internal model has an important additional implication in the hierarchical case, which emerges when we turn to saltatory MB-HRL.

Figure 2*a* (blue) shows the time-course of planning for a second MB-HRL agent, this one furnished not only with doorway options, but also with accompanying option models. Unlike the first MB-HRL agent considered, this new one can perform saltatory MB-HRL: rather than stepping through each primitive-level action and projecting its outcome, the agent can use its option models to leap directly to subgoal locations (see figure 1*c*). This ability to skip over low-level events saves the agent considerable time and computational effort.

The savings associated with saltatory MB-HRL can become crucial in scenarios where the planning agent has highly limited computational capacity. To illustrate this point, figure 2*b* shows the time-course of planning for a set of agents whose ability to simulate long sequences of actions is limited by a tendency to ‘lose their place’ in the planning process (see appendix A). As the figure shows, in this scenario, the difference between saltatory and non-saltatory MB-HRL can mean the difference between success and outright failure. Because saltatory MB-HRL allows the agent to reach the goal via a shorter series of decisions, it allows planning to work even when memory capacity is rather severely restricted. This point is of obvious relevance to the case of human planning, which is dependent upon working memory and other executive resources that are notoriously limited in capacity [21].

The ability to skip over low-level steps, leaping directly to intermediate subgoals, can also make it feasible to plan in situations where an accurate and comprehensive fine-grained model of the environment is unavailable. In such cases, it may be possible to make predictions about the outcomes of temporally extended action sequences, even when the finer-grained events that make up those sequences are difficult to simulate or predict. For example, one may be able to judge quite accurately the probability of defeating a particular opponent in a game of chess, even without the ability to predict what moves that opponent will be prone to make in the game itself (an ability that would clearly be out of reach if the opponent happened to be a superior player).

Another important advantage of saltatory MB-HRL relates to the representation of environmental state. When planning using a primitive-level model, it is necessary to keep track of the current (projected) situation at each step of a candidate action sequence. Depending on the problem domain, this can require tracking detailed features of the environment that may not be important at the level of the overall plan. For example, a detailed simulation of the activity of preparing pasta would require maintaining an explicit record of factors such as whether the sink tap is running, whether the stove is on, whether there is water in the pot and whether that water is boiling. In a saltatory context, where one can simply jump to the conclusion of the activity (a plate of cooked pasta), it may be safe to ignore these transiently important features of the environment, either because execution of the activity will itself assure that they are restored to acceptable defaults (e.g. sink and stove both off), or because they are truly irrelevant to subsequent activities. In saltatory MB-HRL, these points can allow the planning process to attend only to a small core set of environmental features, further lightening the computational load (see [11,22]). A special but important case of such abstraction involves continuous state spaces, where saltatory MB-HRL can permit discretization (see [23]).

A final advantage of saltatory MB-HRL is that it can facilitate ‘backward’ planning, planning that works from ends to means. Because option models immediately represent the expected outcomes of temporally extended behaviours, they make it possible to search among candidate activities based on desired outcomes. If I decide I want to go to a particular cash-only restaurant for dinner but realize that my wallet is empty, knowledge of outcomes allows me to see immediately that a trip to the ATM should be added to my plan (see [24]).

All of these advantages of MB-HRL stem from a common source, which can be understood in information-theoretic terms. The key point is that adaptive naturalistic behaviour carries a high degree of *redundancy*: subsequences of action tend to recur, in differing combinations, across many tasks. Using options to search among candidate behaviours capitalizes upon this redundancy, concentrating search on plans that share structure with previously established routines and are therefore likely to work well [25]. Saltatory MB-HRL goes further, capitalizing not only on the redundancy inherent in adaptive *behaviours*, but also on the redundancy present in *plans* themselves. The option model distils out the few pieces of information that are truly needed for the high-level task, liberating the decision-maker from a profusion of superfluous detail.

Of course, in order to capitalize on the redundancies that pervade adaptive behaviour, it is necessary to first discover those redundancies. There is thus an important learning problem associated with hierarchical control. We will return to this problem in a moment. At the present juncture, however, we turn to the question of whether the computational issues we have been considering are relevant to human action selection.

## 4. Model-based hierarchical reinforcement learning and human planning

Recent empirical studies have provided some evidence supporting the relevance of MF-HRL to human action selection and brain function (see [20]). As a first step in this direction, Botvinick *et al*. [10] reviewed a range of behavioural and neuroscientific findings that appeared consistent with a neural implementation of MF-HRL. Subsequently, neuroimaging data from Ribas-Fernandes *et al*. [26] and Diuk *et al.* [27] have provided support for the occurrence of prediction error signals at both goal and subgoal levels in hierarchical tasks, as occurs in MF-HRL. In parallel work, Holroyd & Yeung [14] have leveraged MF-HRL to build a theory of the role of anterior cingulate cortex in action selection, and Frank & Badre [15] have applied related ideas to prefrontal-basal ganglia circuitry.

What about MB-HRL? While there has been a wealth of research focusing on hierarchical structure in human behaviour [28–33], very little of this work has explicitly engaged the distinction between model-free and model-based processing (although see [34]). Nevertheless, relevant ideas have cropped up intermittently within psychology even since early in its history. William James's [35] theory of volitional action, for example, proposed that prospective, deliberative decision-making occurs only intermittently in the stream of human behaviour and serves primarily to select among and to launch habitized action sequences (see also [36]). Some time later, Tolman [37] advanced a theory of purposive action that posited a hierarchy of superordinate and subordinate goals. Such ideas returned in the 1970s, when interest in hierarchical planning was stimulated by work in artificial intelligence [38,39] (see also [40]).

Despite this history, it is only very recently that experimental studies have begun to offer concrete empirical evidence for hierarchical planning. In these few cases, the data encourage the view that mechanisms from MB-HRL may be relevant. In one study, Huys *et al*. [41] used Bayesian model comparison to provide a detailed analysis of the decision process underlying a multi-step prospective planning task. This revealed a pattern according to which discrete action subsequences were probabilistically ‘reused’ and recombined across planning episodes, consistent with a process whereby model-based action selection operates on chunked or ‘memoized’ subsequences. A similar picture emerges from recent work by Dezfouli & Balleine [42] (see also [43]). This study focused on a multi-step task widely used to study model-based action selection. Based on novel analyses of choice patterns and reaction times, Dezfouli and Balleine concluded that decisions in this task tend to be made not at the level of individual actions but at the level of macro-like action sequences. For example, they observed that when subjects began a trial with the same action that they had used to begin the previous trial, in cases where that previous trial had ended with a reward, subjects were prone to follow up with the same second-step action as well, regardless of the outcome of the first action. And when this occurred, the second action was executed with a brief reaction time, compared to trials where a different second-step action was selected. Working from this and other findings, Dezfouli & Balleine ([42]; see also [12]) proposed a general theory of reward-based decision-making in which a goal-directed (i.e. model-based) process selects among fixed habitual action sequences. ‘Habits’, they write, ‘are learned sequences of actions that, once triggered by the goal-directed process, can be expressed quickly and in an efficient manner…. Habits interact with the goal-directed process in a hierarchical manner; i.e. the goal-directed system selects a goal, and then determines which habit should be executed to reach that goal’ [42, p. 2].

Related evidence has been gleaned from animal experiments. Ostlund *et al*. [44] trained rats on multi-step sequences of action and found that performance of these sequences declined immediately after the resulting food rewards were devalued through satiety. Although this finding was open to more than one interpretation, it was consistent with the possibility that ‘rats can use sequence-level representations, or action chunks, to organize their behaviour in a goal-directed manner’ [44, p. 8280].

Such work provides substantial encouragement for the idea that goal-directed action selection, in humans as well as in other species, may involve mechanisms akin to those involved in MB-HRL. However, what of the distinction between saltatory and non-saltatory MB-HRL? In this review, we have emphasized the special power of saltatory MB-HRL, where option models can be used to ‘skip over’ low-level actions, jumping immediately to option outcomes. Is there evidence that human planners have access to knowledge structures akin to option models and that they leverage them to plan, as in saltatory MB-HRL?

As detailed earlier, option models encode the key pieces of information needed for hierarchical planning, predicting (i) the duration, (ii) the cumulative reward, and (iii) the ultimate outcomes associated with specific temporally extended behaviours. One question that arises naturally from MD-HRL is whether human decision-makers have access to these kinds of summary statistics for familiar activities. Evidence from a number of disparate quarters suggests that this is the case. Concerning cumulative reward—or, equivalently, cumulative cost—there is good evidence that people assign summary evaluations to temporally extended experiences. This point has been a specific focus of inquiry in behavioural economics research, where it has been suggested that such evaluations may arise from applications of heuristic judgement [45–47]. There are also strong indications that people have detailed knowledge concerning the durations of familiar activities. In a pair of studies, Griffiths & Tenenbaum [48,49] provided evidence that people have access not only to mean duration information, but also to probability distributions over duration, as demanded by the option-model construct.^5^

When it comes to outcome prediction, it seems clear from everyday life that people are capable of anticipating, at least coarsely, the ultimate results of temporally extended activities. And as with durations, it seems clear that we can attach probabilities to such outcomes. (One need only think of the forecasts associated with deciding among journals, when preparing to submit a manuscript for review.) Indeed, these abilities are given pride of place in expectancy-value theories of goal selection, which specifically assert that the decision to enter into an activity is determined by probabilistic forecasts of its distal outcomes (e.g. [51]). More immediate evidence for ‘saltation’ in human planning is provided by studies of spatial navigation. Perhaps the most informative study in this regard is by Wiener & Mallot [52]. Here, participants navigated through a virtual environment that was explicitly carved into sharply bounded regions. On critical trials, participants were asked to navigate from one end of the environment to a goal located at the other. The navigation problem was carefully constructed so that there existed two solution paths of equal length. One of these paths crossed two region boundaries, the other only one. Faced with this situation, participants displayed a preference for the path that crossed only a single region boundary. Wiener & Mallot [52] inferred from this choice pattern that navigation planning occurred hierarchically, first targeting transitions between regions, and only subsequently specifying more fine-grained actions within a region.

More direct evidence for saltatory planning comes from recent work by Solway *et al*. [25]. Here, participants were asked to make a series of deliveries within a virtual town, navigating from start to goal locations. The configuration of the town divided individual locations into two neighbourhoods, linked by a single crossroads or ‘bottleneck’ location (akin to the door locations in the rooms domain from figure 2). After participants had gained some experience with making deliveries, they were given new start and goal locations and asked to indicate just one location they would traverse in navigating from one to the other. In response, participants overwhelmingly selected the bottleneck location. On other trials, participants were asked to specify all locations through which their delivery path would pass, but in any order desired. In this situation, the bottleneck location was generally selected first, even when it was not the first location that would be actually traversed. In a further experiment, Solway *et al*. [25] provided evidence that these choice patterns did not simply reflect the greater familiarity of the bottleneck location. Instead, participants appeared to represent the town hierarchically. When asked to navigate from one neighbourhood to the other, they ‘thought first’ of the location linking the two, filling in the finer details of the navigation plan only later. While further research is certainly needed, these findings, along with the others we have briefly reviewed, make it seem likely that the processes underlying human planning can resemble those involved in saltatory MB-HRL.

## 5. The option discovery problem

As intimated in §3, the pay-offs associated with MB-HRL (and indeed, those associated with HRL in general) come with a considerable overhead: in order to enjoy the benefit of options and option models, the decision-maker first has to acquire those knowledge structures through learning. As we have discussed elsewhere [10,20], the hard problem in this context is to discover useful subgoals. In the rooms domain from figure 2, for example, planning is greatly facilitated by access to options leading to the doorway locations. However, this raises the question of how the agent might initially select those locations as useful subgoal destinations.

This learning problem is a major focus of the computational HRL literature, where it has sometimes been referred to as the *option discovery problem*. While a number of approaches have been proposed, some of the most successful are of special interest in the present context because they relate directly to the topic of model-based control. As discussed in §2, a key element of model-based RL is the transition model, an internal representation of action–outcome relationships. A number of interrelated computational studies have proposed that learning the transition model might, in itself, provide a solution to the option discovery problem. One version of this idea involves noticing, based on the transition model, cases where the ability to change certain aspects of the environment depends on other aspects: to open a door, the door must be unlocked; to turn on a television, the television must be plugged in; and so forth. Taking note of such causal dependencies can allow a decomposition of the action space into coherent and useful subtasks [53–56].

**Figure 3.**
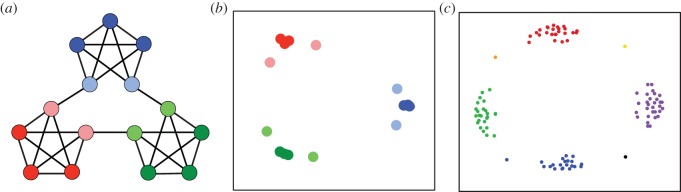
(*a*) The graph employed by Schapiro *et al*. [58]. (*b*) A multidimensional scaling plot, revealing the pattern of similarity among predictive representations of the vertices in the graph. (Adapted from [58].) (*c*) A comparable visualization of successor representations from the rooms domain illustrated in figure 2, with colour indicating aggregation of states using a standard clustering algorithm. The four large clusters correspond to the four rooms. The isolated points correspond to the four doorway locations.

Another way in which model learning might support option discovery is through prediction.^6^ The point can be introduced based on a recent experiment by Schapiro *et al*. [58]. The task in this experiment involved the graph shown in figure 3. Participants never saw this graph. Instead, a unique abstract geometric visual stimulus was assigned to each graph vertex, and the participant viewed a sequence of those stimuli generated based on a random walk through the underlying graph. After an initial period of exposure to this sequential stimulus stream, participants were asked to identify moments in the sequence where they felt that ‘one subsequence had ended and another one begun’. Although participants were told nothing about the underlying transition structure, they showed a tendency to parse the sequence at moments where the random walk traversed one of the bridge-like edges connecting the star-like clusters. Schapiro *et al*. [58] proposed that this segmentation effect arose from an underlying predictive code. Note that vertices that lie within the same star-like cluster overlap in terms of their immediate neighbours. As a result, if each vertex were represented as a vector identifying its potential successors, vertices lying within a cluster would be represented as more similar to one another than vertices lying in different clusters. Thus, if participants in the experiment represented each visual stimulus partially in terms of which other stimuli it predicted, this would have provided an opportunity to discover the clustered structure of the overall domain (see figure 3). Schapiro *et al*. [58] gleaned evidence for this hypothesis using functional neuroimaging: fMRI conducted during viewing of the stimuli from the experiment revealed the predicted pattern of representational similarity within regions of the inferior frontal and anterior temporal cortices.

The theory put forth by Schapiro *et al*. [58] translates directly into an account of how building a transition model might support subgoal discovery. To illustrate this, we return once more to the rooms domain from figure 2. We allow an RL agent to learn a transition function through ‘latent learning’ [37], that is, by simply exploring the rooms grid, observing the outcomes of its actions. From this transition function, we allow the agent to compute a *successor representation* (see [59]), a representation of each state (location) in terms of the other states it predicts will soon be visited. The resulting representation of the start location, for example, indicates that when the agent is in this location, it can be expected soon to occupy one of the nearby locations in the same room. As shown in figure 3, the similarities among the full set of such representations immediately reveal the overall organization of the rooms domain, highlighting the doorway locations. In this sense, prediction-based representation offers a platform for identifying useful subgoals. Again it appears that, while MB-HRL raises the problem of option discovery, model learning itself may provide a path towards solving that problem.

## 6. Conclusion

We have considered the intersection between two forms of RL, both of which have attracted recent interest in cognitive science and neuroscience, but whose interrelations have not yet been widely considered in those fields. Model-based RL is defined by the use of an internal model of the environment to perform prospective planning. Hierarchical RL, in turn, is defined by the use of temporally abstract actions or options. Lying at the intersection of these two paradigms is the field of model-based hierarchical RL.

The points we have considered suggest that it may be productive to further investigate the potential relationship between MB-HRL and human action selection. We see two broad motivations for this view. The first is related to the dramatic computational leverage MB-HRL offers, which, as we have seen, can make planning feasible in settings where it otherwise might fail. Given the complex multi-step problems human decision-makers face in everyday life, and the apparent ease with which these are often negotiated, it seems appealing to consider whether human decision-making might leverage some of the very computational tools that are involved in MB-HRL. The second and more immediate motivation for continued inquiry into the psychological relevance of MB-HRL lies in recent evidence, briefly reviewed above, suggesting that related mechanisms might indeed be at work in both human and rodent decision-making.

In discussing pertinent data, we have focused primarily on behavioural findings. In future research, it will be interesting to consider potential neural correlates for MB-HRL. Intriguingly, functional neuroimaging studies have implicated overlapping regions within the dorsolateral prefrontal cortex in model-based control [4,60] and hierarchical action representation [61,62] (although see [63–65]). Also potentially relevant is the recent discovery that medial temporal lobe structures including the hippocampus may be critical for projecting future events, a critical component of planning [66], an idea consistent with the longer standing view that the hippocampus carries a ‘cognitive map’ which supports spatial navigation [67]. In this connection, it is interesting to note that the representational similarity effects reported by Schapiro *et al*. [58], reviewed in §5, have subsequently been noted by the same investigators also to appear within hippocampus (unpublished data). This finding suggests that the same neural mechanisms that underlie the projection of future events may also be sensitive to hierarchical structure in those events.

Cognitive and neuroscientific studies focusing on model-based RL have highlighted the question of arbitration: if the brain contains systems for performing both model-free and model-based action selection, how is it decided which of these systems controls behaviour at any given moment [3]? What factors determine the balance of power between the two systems? Are they necessarily in competition, or do they perhaps work together in some ways [5]? These questions arise, with equal force, in the context of HRL. As we have noted, available evidence suggests that both model-free and model-based forms of HRL may be at work in human decision-making. If so, what is the functional relationship between these? Do they collaborate? Compete? Do they encode the same hierarchical relationships or are there settings in which their representations of behaviour may differ [34]?

The intrinsic interest of such questions, together with the encouraging results of initial research, suggests that MB-HRL may prove useful as a source of hypotheses and predictions for the next phase of research on reward-based decision-making.

## Endnotes

1 Technically, the term *model-based* applies to any form of reinforcement learning that makes use of a domain model. This includes learning algorithms (e.g. temporal difference learning with a state-value function) that depend on direct interaction with the environment, rather than planning. However, in recent work in cognitive science and neuroscience, *model-based reinforcement learning* has typically been used as a synonym for reward-based planning, and this is the usage we adopt here.

2 The sequence of actions involved here is typically ‘closed loop’, in the sense that the actions selected depend on the outcomes encountered after each action. However, some theories have also considered ‘open-loop’ sequences, where actions are chosen without regard to environmental state (e.g. [12]). It is also worth noting that the actions in question may include not only overt motor behaviours, but also ‘cognitive actions’ governing attention, memory and other faculties (see [13]).

3 The options framework is one of several successful implementations of HRL. For a discussion of other schemes, see [9].

4 In order to accomplish this, each option is also furnished with its own value function. For details, see [10,16].

5 As in the case of cumulative reward, other research suggests that estimates of duration may be subject to systematic biases or distortions (e.g. optimistic underestimation of how long it will take to complete future activities; e.g. [50]). It should be noted that such distortions are not incompatible with MB-HRL, because it is nowhere stipulated in the MB-HRL framework that the agent's internal model must necessarily be accurate. At the same time, it should also be noted that, even when the model is inaccurate, it may still be of use due to the enormous computational savings that arise from temporal abstraction.

6 There is, in fact, a fundamental connection between option discovery based on prediction and option discovery based on causal dependencies. For a discussion of the relationship between the two, see [57].

## Appendix A

Here, we briefly describe the methods employed in the simulation illustrated in figure 2.

The flat RL agent was implemented using a standard actor–critic architecture, following the same approach as described in Botvinick *et al*. [10], and using the parameter values specified in that source. In order to realize the actor–critic as a model-based implementation, the base model was placed in series with a generative model of the environment, considered as lying internal to the agent. Action outcomes were viewed as coming from this model, rather than from the environment itself (see [8,68,69]).

In order to implement MB-HRL, we extended the actor–critic architecture to accommodate options, as described in Botvinick *et al*. [10], again using the same parameter values. Once again, the set-up was treated as model-based in the sense that action outcomes were viewed as coming from the agent's internal transition model, rather than from the environment itself. In order to implement saltatory MB-HRL, the same simulation code was employed, but the count of steps taken was not incremented during execution of option policies. This approach simulated a scenario in which the agent had an accurate model for each option and sampled from its transition, duration and cost distributions in generating plan trajectories.

To implement memory limitations, it was assumed that each agent had a 10% chance on each step of ‘losing track’ of which state was to be sampled. This event was understood as triggering an immediate termination of the planning trial and initiation of the next trial. Note, however, that the data in figure 2 are intended to illustrate performance on each trial had the trial continued without interruption. To accomplish this, rather than literally terminating trials when forgetting occurred, the learning rate was instead set to zero for the remainder of the trial and the trial continued until the goal was reached.
